# Quality control of the sheep bacterial artificial chromosome library, CHORI-243

**DOI:** 10.1186/1756-0500-3-334

**Published:** 2010-12-13

**Authors:** Abhirami Ratnakumar, Ewen F Kirkness, Brian P Dalrymple

**Affiliations:** 1 CSIRO Livestock Industries, 306 Carmody Road, St. Lucia, QLD 4067, Australia; 2 Department of Medical Biochemistry and Microbiology, Uppsala University, Box 582, 751 23 Uppsala, Sweden; 3 J. Craig Venter Institute, 9704 Medical Center Drive, Rockville, MD20850, USA

## Abstract

**Background:**

The sheep CHORI-243 bacterial artificial chromosome (BAC) library is being used in the construction of the virtual sheep genome, the sequencing and construction of the actual sheep genome assembly and as a source of DNA for regions of the genome of biological interest. The objective of our study is to assess the integrity of the clones and plates which make up the CHORI-243 library using the virtual sheep genome.

**Findings:**

A series of analyses were undertaken based on the mapping the sheep BAC-end sequences (BESs) to the virtual sheep genome. Overall, very few plate specific biases were identified, with only three of the 528 plates in the library significantly affected. The analysis of the number of tail-to-tail (concordant) BACs on the plates identified a number of plates with lower than average numbers of such BACs. For plates 198 and 213 a partial swap of the BESs determined with one of the two primers appear to have occurred. A third plate, 341, also with a significant deficit in tail-to-tail BACs, appeared to contain a substantial number of sequences determined from contaminating eubacterial 16 S rRNA DNA. Additionally a small number of eubacterial 16 S rRNA DNA sequences were present on two other plates, 111 and 338, in the library.

**Conclusions:**

The comparative genomic approach can be used to assess BAC library integrity in the absence of fingerprinting. The sequences of the sheep CHORI-243 library BACs have high integrity, especially with the corrections detailed above. The library represents a high quality resource for use by the sheep genomics community.

## Findings

We have recently demonstrated for the bovine CHORI-240 BAC library that it is possible to identify BACs with confused identities using three independent datasets (such as genome sequences, BESs and BAC fingerprints) [1]. BACs whose identities are not consistent across the three datasets are likely to have been confused, missed, duplicated or misassigned somewhere in the generation, copying or use of the library. The sheep CHORI-243 BAC library [2] will be important for the assembly of the sheep genome, particularly for the reference genome assembly based on the animal used to construct the BAC library. Hence it is extremely important to determine the integrity of the BAC-end sequences, the clones and the plates in the library. In contrast to the bovine CHORI-240 library, for the sheep BACs three or more independent datasets are not available. Here, we have addressed the question, is it possible to identify BACs with issues in a library where only the BESs themselves have been determined? To do this we used the virtual genome sequence, which is a reordering of the bovine genome sequence into the predicted order of the sequence in sheep using the integrated mapping of sheep BACs to a number of genome sequences [2,3].

## Plate biases identified by sequence alignments

**Figure 1:**
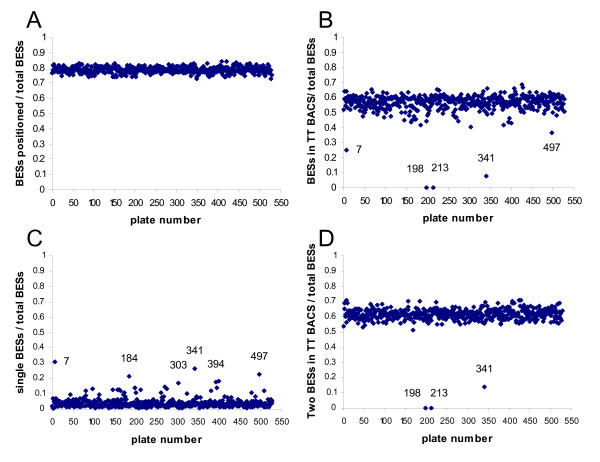
**Proportion of the total number of sheep BESs for each plate of the BAC library**. A. positioned on the virtual sheep genome. B. positioned in tail-to-tail (TT) BACs. C. in BACs with only one end positioned on the virtual sheep genome. D. Proportion of the total number of sheep BACs with two end sequences and tail-to-tail organisation for each plate of the BAC library.

The proportion of the total number of BESs from each CHORI-243 plate positioned on the virtual sheep genome assembly v2.0 [3] was calculated (Figure 1A). In general a very consistent proportion of BESs from each plate was positioned, indicating that there were few plate biases identified using the alignment process.

A more sensitive measure of integrity is to look at the number of BACs mapped to the virtual sheep genome with their BESs in a tail-to-tail arrangement, i.e. concordant (the organisation of the original sheep sequence in the sheep genome). If the integrity of the identity of reads is maintained the proportion of BACs with tail-to-tail paired end reads should be roughly constant across all plates. On the basis of the BES positions in the bovine genome the BACs were grouped into concordant BACs (tail-to-tail), discordant BACs (i.e. tail-to-tail outsize, tail-to-head etc.). A plot of the proportion of tail-to-tail BACs versus the total number of BACs per plate that were positioned, revealed some plates (7, 213, 198, 341 and 497) likely to have experienced problems in the end sequencing (Figure 1B). Plates 213 and 198 had no tail-to-tail BACs at all. However, plate 7 also had a large number of BACs with only one end sequenced (Figure 1C). Plotting the proportion of tail-to-tail BACs to BACs with sequences from both ends of the BAC (Figure 1D) identified that three plates, 198, 213 and 341 were definitely problematic. Further analyses revealed that some BAC-end sequences from BACs on plate 198 appear to have been derived from BACs on plate 213 and vice versa. However, a straight swap of the SP6 or T7 BESs between plates 198 and 213 only increased the number of tail-to-tail BACs to 67 for each of the plates, which is still well below the expected value of 183 BACs based on the dataset average. Thus the problem appeared to be more complicated than a straight swap of BESs between the two plates during sequencing. Plate 341 was also investigated in more detail (see below).

## BES and BAC overlaps

**Figure 2:**
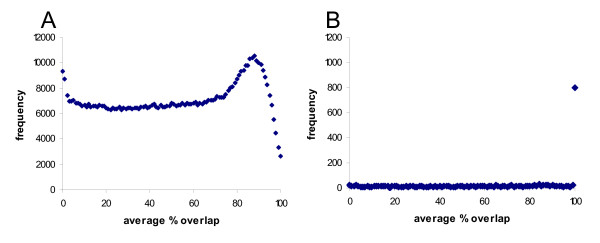
**The distribution of percent overlaps of BACs in the virtual sheep genome**. A. tail-to-tail sheep BACs on different plates in the sheep BAC library. B tail-to-tail BACs on the same plate in the sheep BAC library.

During the assembly of genomes the number of links between different segments of DNA in contigs and scaffolds is frequently a key factor in ordering and orientating the segments. It is important that these linkages are independent. In our previous analysis of a cow BAC library we observed a bias towards BACs with more than 75% overlapping regions of genome coverage [1]. The average percent overlap of tail-to-tail sheep BACs overlapping with a tail-to-tail sheep BAC on a different plate in the CHORI-243 library (Figure 2A), or overlapping with another tail-to-tail sheep BAC on the same plate in the CHORI-243 library (Figure 2B) was determined. For overlapping BACs within the same plate in the library the major peak is at 100% overlap. The plot of overlaps between different plates in the library shows the major peak at 88% overlap. This supports the proposal that overlapping BACs present on the same plate in the library should not both be used in the construction of genome assemblies as there is a high probability that they are not independent clones.

**Figure 3:**
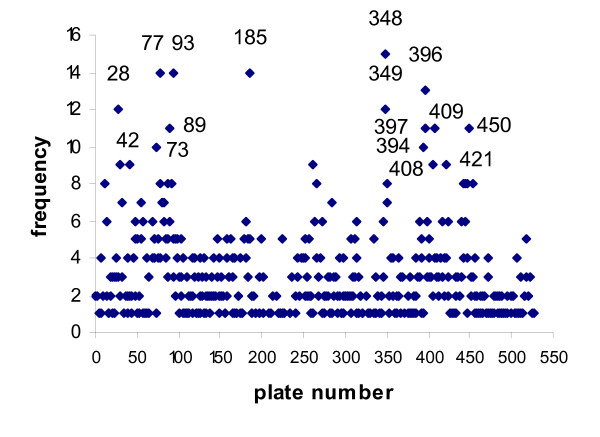
**Overlapping BESs**. The frequency of BACs with at least 1 BES overlapping with the BES of a different BAC on the same plate that is located 0, 1 or 2 wells apart either on the same row or same column. To improve clarity the data from plate 341, which had 127 cases, was not included in the plot.

A plot of the frequency of sheep BACs on the same plate in the CHORI-243 library with overlapping BESs in wells on the same row or column, and 1 or 2 wells apart, revealed that there are a subset of library plates that show a much higher than average frequency of these patterns (Figure 3). This indicates possible localised cross contamination within library plates with the same BAC. In particular plate 341 had 127 such cases (data not included on Figure 3). This supports the more general proposal that BACs with an overlapping BES present on the same plate in the library should not both/all be used in the construction of genome assemblies as there is a high probability that they are not independent clones.

## Plate 341

In the analyses of the proportion of tail-to-tail BACs versus total BACs for each plate (Figure 1B), plate 341 was highlighted as having a much lower proportion than all but two other plates. In addition, of the 4,330 pairs of BACs with at least 1 BES overlapping with the BES of a different BAC on the same plate 2,257 pairs were on plate 341. Since this number substantially exceeds the number of BACs on the plate this suggests that many of the overlapping clones overlapped with multiple BESs from this plate. Plate 341 also has the highest frequency of BES overlaps on the same plate that are in the same row or column and 1 or 2 wells apart (127).

On closer examination it was found that 59 BESs from plate 341 mapped to the same 33 base region on BTA2, that there were 29 different BESs that mapped to the same 35 base region on BTA3 and a further different set of 13 BESs that mapped to a 106 base region on the unknown chromosome fragment BTAUn.2926. All of the BESs in these groups had been determined using the SP6 sequencing primer. The most likely explanation appeared to be a contaminating segment of DNA, probably not of sheep origin that had been sequenced from many but not all SP6 primer samples taken from the original 384 well plate. Searching the GenBank non-redundant nucleotide sequence database identified the *Nitrosococcus oceani *16S rRNA gene as the most likely source of the contaminating sequence. The matches of these sequences to the bovine genome appear to have been fortuitous and do not represent similar contamination of the bovine genome sequence. All sheep BES entries on plate 341 that appear to contain the contaminating sequence have been removed from GenBank.

## Other issues

The observation of significant contaminating 16 S rRNA sequences on plate 341 prompted us to look at across the rest of the BESs for similar issues. A small number of additional BESs, the SP6 reads from BACs CH243-111E1 (DU370043), CH243-111E6 (DU370458), CH243-111E17 (DU370050), CH243-111G6 (DU370470), CH243-111K23 (DU370085) and CH243-338D4 (DU483159) also appeared to be derived from bacterial 16 S rRNA sequences or chimeras between real sheep sequence and bacterial 16 S rRNA sequences. These entries have now been removed from GenBank.

## Conclusions

Evaluation of the sheep CHORI-243 library was undertaken without BAC fingerprinting data, or the availability of a sheep genome assembly. Despite this, sequence comparisons which used BESs allowed for a detailed search for technical errors which may have occurred during library construction, replication and sequencing. Comparison with the CHORI-240 library from cattle indicates the sheep resource contains many fewer cases of loss of integrity. We have demonstrated that comparative genomics can be used effectively to maximise the numbers of BACs able to be analysed. However, using high sensitivity search conditions to maximise alignment of the sheep sequences initially actually reduced our ability to identify contaminants with non-sheep sequence, as demonstrated by the failure to identify plate 341 as a problem just on alignment rates alone, despite the almost 200 BESs that were determined from a contaminating segment of bacterial 16 S rRNA DNA.

Further comparison of the sheep and cow analysis results show that the distributions of BAC overlap within plates is very similar, in both there is a strong bias towards complete and almost completely overlapping BACs. In the case of overlaps between BACs on different plates the peaks are both at around 90% overlap, although one was calculated from restriction enzyme fingerprints and the other from mapping of BESs to a related genome. Within the same plates overlaps are likely to be due to the same physical clone being analysed twice. In contrast, very few, if any, overlaps between clones on different plates are predicted to be due to exactly the same clone. Overall the sheep BACs exhibited less bias towards large overlaps than the bovine BACs, but whether this reflects differences in the library construction, differences in the genome structure, or in the virtual sheep genome construction process is not clear.

These analyses show that the integrity of the sheep BESs in the CHORI-243 library is very high. In addition, we have demonstrated in a simplification of our previous methodology that the integrity of paired end reads from long insert clones can be validated using comparative genomics approaches in the absence of fingerprinting. Thus the methods that we have described are very broadly applicable.

## Abbreviations

BAC: Bacterial Artificial Chromosome; BES: BAC end sequence; Vsg2.0: virtual sheep genome version 2.0.

## Competing interests

The authors declare that they have no competing interests.

## Authors' contributions

AR carried out the majority of the analysis and helped to draft the manuscript. EK, undertook some of the analysis, BPD conceived, designed and coordinated the study and drafted the manuscript. All authors read and approved the final manuscript.
